# Preclinical Evaluation of an Activity-Based Probe for Intraoperative Imaging of Esophageal Cancer

**DOI:** 10.1155/2022/5447290

**Published:** 2022-07-14

**Authors:** Gregory T. Kennedy, Feredun S. Azari, Bilal Nadeem, Ashley Chang, Alix Segil, Elizabeth Bernstein, Charuhas Desphande, John C. Kucharczuk, Edward J. Delikatny, Sunil Singhal

**Affiliations:** ^1^Department of Surgery, University of Pennsylvania School of Medicine, Philadelphia, PA, USA; ^2^Department of Pathology, University of Pennsylvania School of Medicine, Philadelphia, PA, USA; ^3^Department of Radiology, University of Pennsylvania School of Medicine, Philadelphia, PA, USA

## Abstract

**Background:**

Early detection and complete resection are important prognostic factors for esophageal cancer (EC). Intraoperative molecular imaging (IMI) using tumor-targeted tracers is effective in many cancer types. However, there are no EC-specific IMI tracers. We sought to test a cathepsin activity-based tracer (VGT-309) for EC resection.

**Methods:**

Murine (AKR, HNM007) and human (OE19) EC cell lines were screened for cathepsin expression by western blotting. *In vitro* binding affinity of VGT-309 was evaluated by fluorescence microscopy. Flank tumor models were developed by injecting EC cells into the flanks of BALB/c or athymic nude mice. Mice pretreated with a cathepsin inhibitor (JPM-OEt) were used to confirm on target binding. Animals were injected with 2 mg/kg VGT-309, underwent IMI, and were sacrificed 24 hours after injection.

**Results:**

Cathepsins B, L, S, and X were expressed by EC cell lines, and all cell lines were labeled *in vitro* with VGT-309. Fluorescent signal was eliminated when cells were pretreated with JPM-OEt. On biodistribution analysis, VGT-309 accumulated in the liver, kidneys, and spleen without other organ involvement. VGT-309 selectively accumulated in flank allografts and xenografts, with mean signal-to-background ratio of 5.21 (IQR: 4.18-6.73) for flank allografts and 4.34 (IQR: 3.75-5.02) for flank xenografts. Fluorescence microscopy and histopathological analysis confirmed the selective accumulation of the tracer in tumors compared to background normal tissues.

**Conclusions:**

VGT-309 is an effective tracer for IMI of esophageal cancer. There is potential for clinical translation both as an adjunct to endoscopic detection and for complete removal of disease during esophagectomy.

## 1. Introduction

Esophageal cancer is the eighth most common cancer worldwide, with an annual global incidence of approximately 450,000 [1]. The epidemiology of the disease is rapidly evolving; historically, esophageal squamous cell carcinoma (ESCC) was the predominant histologic subtype, but there has been a dramatic rise in the proportion of patients with esophageal adenocarcinoma (EAC). The burden of disease is growing with some estimates predicting that by 2030, one in 100 men may be diagnosed with esophageal cancer in Western countries [2]. The dominant prognostic factor for survival is complete (R0) surgical resection with negative margins. However, a substantial proportion of patients suffer from recurrence after curative-intent resection, due to incomplete clearance of disease at the index operation [3].

Intraoperative molecular imaging (IMI) has been developed as a method to improve the likelihood of achieving negative margins during oncologic surgery. IMI involves delivering tumor-targeted optical contrast agents to cancer cells and detecting them with wavelength-specific camera systems during surgery [4]. This approach has been applied to detection of cancerous tissue *in vivo* as well as back table evaluation of specimens to guide identification of tumor-positive margins [citations]. IMI has been proven effective in the resection of many solid tumors including ovarian [5], lung [6–8], mesothelioma [9, 10], brain [11], and head and neck cancers [12, 13], but there are no IMI tracers for esophageal cancer [14]. Thus, there is a pressing, unmet need for the development of esophageal cancer-targeted tracers that can aid surgeons in detecting occult disease during resection.

A promising enzyme target family for IMI tracers for esophageal cancer is the cysteine cathepsins [15]. Cysteine cathepsins are a class of proteases that have established functions as enzymes of the lysosomal degradation system [16]. Cathepsin protease activity is frequently dysregulated in the context of neoplastic transformation, and increased cathepsin expression has been demonstrated in esophageal tumors [16]. Aberrant activity and localization of cysteine cathepsins within the tumor microenvironment plays a potent role in driving cancer progression, proliferation, invasion, and metastasis [17]. Cathepsins, including cathepsins B and L, have been shown to be highly expressed in esophageal tumors [18, 19], but their expression is limited in normal esophageal and gastric mucosa, making them an ideal esophageal cancer imaging target [20].

In this study, we evaluated VGT-309, a cathepsin-targeted IMI probe, for the intraoperative detection of esophageal cancer. VGT-309 is a covalent quenched activity-based probe (qABP), meaning that it becomes fluorescent after covalently binding cysteine cathepsins. The probe uses the fluorophore ICG, which can be detected by FDA-approved surgical imaging devices that are widely available in clinical practice. Here, we conducted a comprehensive preclinical evaluation of VGT-309, including cellular and animal models of EAC and ESCC. We found that VGT-309 selectively labeled tumor cells at both macroscopic and microscopic levels with minimal background fluorescence. To our knowledge, this is the first study of a covalent qABP for labeling esophageal cancer during resection.

## 2. Methods

### 2.1. Study Drug

**Figure 1:**
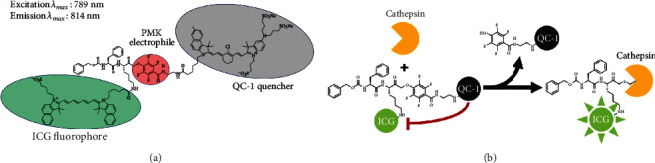
Structure, optical properties, and mechanism of action of VGT-309. (a). VGT-309 consists of a cathepsin-targeted PMK electrophile coupled to an ICG fluorophore and QC-1 quencher. Its maximum absorption and emission wavelengths are 745 nm and 820 nm, respectively. (b). Mechanism of action of VGT-309. The ICG fluorophore is quenched by the QC-1 quencher until it is cleaved upon covalent binding by cysteine cathepsins.

VGT-309 (chemical formula C_127_H_142_ClF_4_N_10_Na_3_O_23_S_5_; molecular weight, 2517.29 Da) is a quenched activity-based probe (qABP) that consists of a phenoxymethyl ketone electrophile that covalently and irreversibly binds active cysteine cathepsins, coupled to a ICG fluorophore (Figure 1(a), excitation peak = 789 nm, emission peak = 814 nm) and a IRDye QC-1 quencher (LI-COR Biosciences, Lincoln, NE). Upon binding, the quencher is released to allow NIR fluorescence signal from the probe (Figure 1(b)). The cysteine cathepsins that have been shown to be targeted by VGT-309 include cathepsins B, L, S, and X [21]. Given that VGT-309 binds active cathepsins and cathepsins have been found to function both intracellularly and extracellularly in tumors, it is thought that VGT-309 labels cathepsins both inside and outside the cell [22]. VGT-309 drug substance and drug product were manufactured in compliance with Good Manufacturing Practices by LI-COR Biosciences (Lincoln, NE) and UI Pharmaceuticals (Iowa City, IA), respectively. Vials containing 11 mg of lyophilized VGT-309 drug product were reconstituted with water for injection to a final concentration of 5 mg/mL and diluted with pH 7.4 phosphate-buffered saline, culture media, or Baxter Compound Sodium Lactate (Hartmann's) for the appropriate application.

### 2.2. Cell Lines

The human cervical carcinoma cell line KB was chosen as a positive control in our experiments, given its previous use as a control in studies of cathepsin expression in human tumors [23]. A range of human and murine esophageal cancer cell lines were obtained from the ATCC or from collaborators at the University of Pennsylvania. These included OE19 (human esophageal adenocarcinoma), AKR (murine esophageal squamous cell carcinoma), and HNM007 (murine esophageal squamous cell carcinoma). Cell lines were maintained *in vitro* using media containing RPMI, 10% fetal bovine serum (FBS), 2 mmol/L glutamine, and 5 mg/mL penicillin/streptomycin. Cell lines were regularly tested and maintained negative for *Mycoplasma* spp.

### 2.3. Western Blotting Assays

Preparation of whole cell protein lysates and western blotting were performed as described previously [24]. Briefly, whole cell protein lysates (50 *μ*g) were electrophoresed through 12% denaturing polyacrylamide slab gels, and the protein bands were transferred to a polymer of vinylidene fluoride (PVDF) membrane (Bio-Rad, Hercules, CA, USA) by electroblotting. Primary antibodies against cathepsins B (Santa Cruz Biotechnology, catalog number sc-365558), L (sc-32320), S (sc-271619), and X (sc-376976) were used in our study. These cathepsins were studied given prior work showing binding of VGT-309 to these cysteine cathepsins [21].

### 2.4. SDS-PAGE Analysis of Probe-Labeled Species

To evaluate the binding specificity of VGT-309 to individual cysteine cathepsins as well as the activity dependence of the probe, we conducted fluorescent SDS-PAGE analysis of probe-labeled species using a protocol previously described [25]. Briefly, we incubated OE19 cells with 1 *μ*M VGT-309 for 2 hours after pretreatment of cells for 30 minutes with 100 *μ*M JPM-OEt (MedChemExpress, Monmouth, NJ), a pan-cathepsin inhibitor. We lysed the cells and loaded lysates and a fluorescent molecular weight maker on an SDS-PAGE gel and ran the gel for 15 minutes at 80 V and subsequently at 130 V until the dye front had run off the gel. Gels were imaged on the Odyssey NIR scanner (LI-COR Biosciences, Lincoln, NE) and individual cathepsin bands were assigned by molecular weight. To control for equal protein loading, gels protein bands were transferred to a PVDF membrane and probed for GAPDH (Santa Cruz Biotechnology) as a loading control.

### 2.5. Evaluation of In Vitro Binding and Internalization of VGT-309 in Human Esophageal Cancer Cells by Fluorescence Microscopy

Cell lines were cultured on poly-L-lysine-coated glass coverslips in 6-well plates with RPMI media supplemented with 10% FBS, L-glutamine, and penicillin/streptomycin for 24 hours. For internalization studies, cells were treated with 200 nM LysoTracker (Invitrogen, Waltham, MA) for 2 hours prior to incubation with 1 *μ*M VGT-309. Coverslips were removed from culture at 60 minutes following VGT-309 treatment. For cathepsin inhibition experiments, cells underwent a 30-minute pretreatment with 100 *μ*M JPM-OEt (MedChemExpress, Monmouth, NJ), a pan-cathepsin inhibitor. Pretreated and non-pretreated cells were then incubated with 1 *μ*M VGT-309 for 1 hour. Following dye administration, coverslips were mounted on glass slides with ProLong Gold Antifade reagent with DAPI (Fisher Scientific, Waltham, MA) and imaged on a Leica DM6 B fluorescence microscope (Leica Microsystems, Wetzlar, Germany).

### 2.6. Evaluation of In Vivo Tumor Labeling in Small Animal Models

Female BALB/c mice bearing flank AKR and HNM007 allografts (*n* = 3 per cell line) and female nude athymic mice bearing OE19 flank xenografts measuring 250 ± 50 mm^3^ were intravenously administered VGT-309 at a dose of 2 mg/kg. As a negative control, mice bearing KB flank xenografts were administered 50 mg/kg JPM-OEt via intraperitoneal injection daily for five days prior to VGT-309 administration. As a positive control, mice bearing KB flank xenografts were administered the JPM-OEt delivery vehicle without the cathepsin inhibitor via intraperitoneal injection daily for five days prior to VGT-309 administration. Twenty-four hours after VGT-309 injection, mice were euthanized and imaged with the Iridium Imaging System (Vision Sense, New York, NY). Tumors were removed and imaged with the Odyssey Imaging System (LI-COR Biosciences, Lincoln, NE). Tissue sections were further analyzed by hematoxylin and eosin staining, as well as fluorescence microscopy (Leica Microsystems, Wetzlar, Germany).

### 2.7. Histopathologic and Fluorescent Microscopic Review of Specimens

Specimens were formalin fixed and paraffin embedded. Sequential 5*-μ*m sections were obtained and underwent comprehensive histopathologic and fluorescent analysis by a board-certified thoracic pathologist. Sections were stained using standard hematoxylin/eosin (H&E) staining. To understand VGT-309 accumulation patterns at a microscopic level, an additional unstained 5*-μ*m section was evaluated using a NIR microscopic scanner (Odyssey, LiCor, Lincoln, NE) and a NIR microscope (Leica Microsystems, Buffalo Grove, IL). Areas of fluorescence were then correlated to the H&E specimens.

### 2.8. Post Hoc Image Analysis

Post hoc image analysis was conducted with ImageJ (http://rsb.info.nih.gov/ij). Mean fluorescence intensity (MFI) of the lesion (MFI_lesion)_ was obtained by analyzing monochromatic NIR images and measuring the region of interest which correlated to the lesion. Background fluorescence (0.5–1.0 cm from margin) was also obtained (MFI_background_). Calculations were repeated in triplicate, and signal-to-background fluorescence ratios (SBR) were calculated using the following equation (MFI_lesion_/MFI_background_). SBR assessments were averaged, and a mean SBR>2.0 was considered fluorescent.

### 2.9. Statistics

For *in vitro* assays and *in vivo* murine studies, at least 3 samples were utilized per group unless noted. *Post hoc* image analysis was performed to quantify the amount of fluorescence at the location of the tumor using ROI software within ImageJ (NIH; https://imagej.nih.gov/ij). A background fluorescence level was similarly obtained, and signal-to-background ratio (SBR) was calculated. Pearson coefficient of colocalization of fluorescent signal was assessed using the Coloc2 plugin in ImageJ. Results are expressed as mean (SD) unless otherwise noted. All comparisons were made using Stata Statistical Software release 14 (StataCorp, College Station, TX). A *p* value of 0.05 or less was considered statistically significant.

## 3. Results

### 3.1. Esophageal Cancer Cells Express Cathepsins That Are Fluorescently Labeled by VGT-309

**Figure 2:**
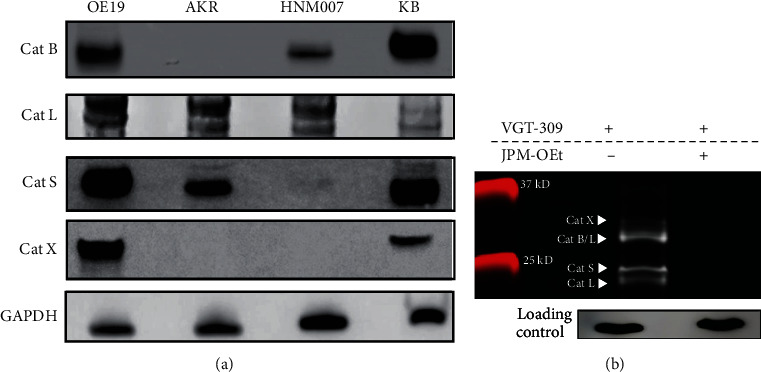
Esophageal carcinoma cell lines express cathepsins that are fluorescently labeled by VGT-309. (a). Western blot analysis of cathepsin expression in esophageal cancer cell lines with KB (human cervical carcinoma) used as a positive control. (b). SDS-PAGE analysis of probe-labeled species in OE19 (human esophageal adenocarcinoma) cells with and without pretreatment with 100 *μ*M JPM-OEt, a broad spectrum cathepsin inhibitor.

To study the ability of VGT-309 to label esophageal cancer at the cellular level, we first performed western blot analysis to confirm cathepsin expression in three murine and human esophageal cancer cell lines of varying histopathological subtypes (Figure 2(a)). These included OE19 (human esophageal adenocarcinoma), AKR (murine esophageal squamous cell carcinoma), and HNM007 (murine esophageal squamous cell carcinoma). The human cervical carcinoma cell line KB was chosen as a positive control in our experiments, given its previous use as a control in studies of cathepsin expression in human tumors [23]. To evaluate the binding specificity of VGT-309 to individual cysteine cathepsins as well as the activity dependence of the probe, we incubated OE19 cells with 1 *μ*M VGT-309 for 2 hours after pretreatment of cells for 30 minutes with 100 *μ*M JPM-OEt, a pan-cathepsin inhibitor. This inhibitor binds irreversibly in the active site of the cysteine cathepsins, thus blocking the binding of VGT-309. We collected and lysed these cells and ran the lysates on an SDS-PAGE gel to examine individual cathepsins by molecular weight. We found that VGT-309 labeled all examined cathepsins, and that pretreatment with JPM-OEt blocked labeling of all cathepsins, confirming the high degree of selectivity of the probe (Figure 2(b)).

**Figure 3:**
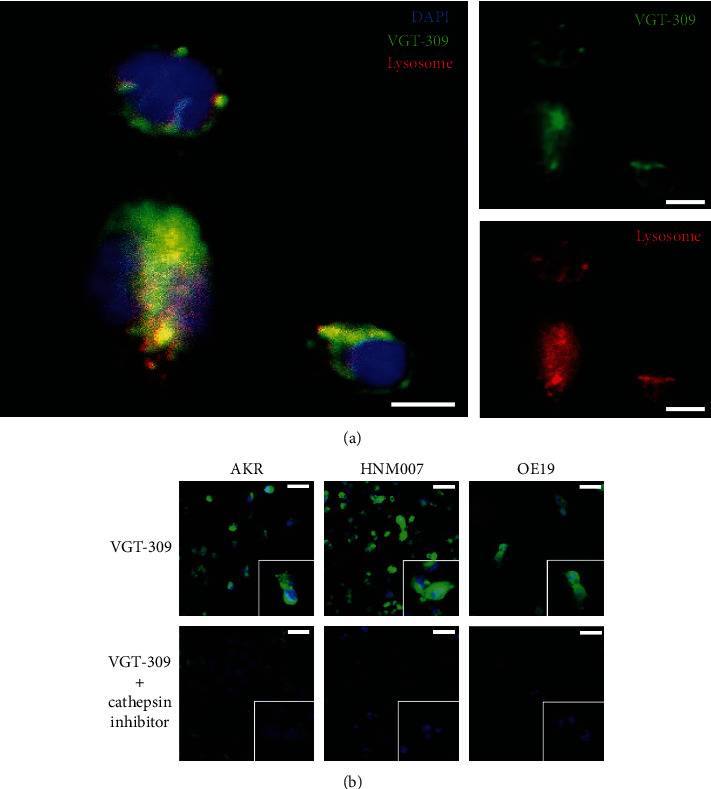
VGT-309 labels esophageal cancer cell lines in a cathepsin activity-dependent manner. (a). Fluorescence microscopy of OE19 (human esophageal adenocarcinoma) cells at 100× magnification 1 hour after treatment with 1 *μ*M VGT-309. Cells were costained with DAPI and LysoTracker. VGT-309 labeling (shown in green) is predominantly lysosomal (shown in red) in distribution. Overlay images are shown at left with VGT-309 channel and LysoTracker channels shown at right. Scale bars represent 5 *μ*m. (b). Fluorescence microscopy at 10× and 40× (inset images) showing VGT-309 fluorescence in esophageal cancer cells with and without pretreatment with JPM-OEt, showing cathepsin activity dependence of fluorescence. Scale bars represent 20 *μ*m.

To assess the localization and internalization of VGT-309, we imaged live OE19 cells at 1 hour after staining with 1 *μ*M VGT-309 and 200 nM LysoTracker. We found that VGT-309 freely entered cells and reacted with active cysteine cathepsins to unquench the probe. Fluorescent signal was primarily lysosomal in distribution (Figure 3(a), Pearson coefficient = 0.83). To assess the cathepsin activity dependence of cell labeling, we incubated all cell lines with VGT-309 and imaged them with fluorescence microscopy at 1 hour. There was near total elimination of signal in cells pretreated with JPM-OEt, indicating the cathepsin activity dependence of fluorescent signal (Figure 3(b)).

### 3.2. VGT-309 Fluorescently Labels Esophageal Cancer Flank Tumors in Syngeneic and Xenograft Mouse Models

**Figure 4:**
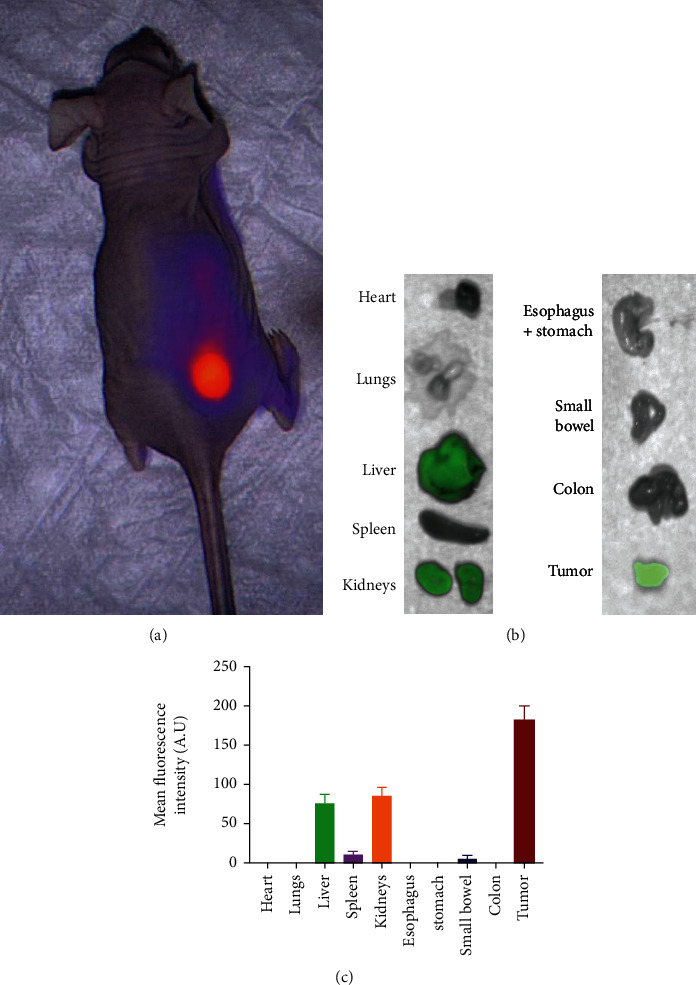
Biodistribution of VGT-309 in mice. (a). Mice bearing OE19 flank allografts were administered 2 mg/kg VGT-309 and imaged using the Iridium NIR imaging system. (b). Twenty-four hours after delivery of VGT-309 at 2 mg/kg, mice were euthanized to determine drug biodistribution. Fluorescence of organs and tumors were obtained using the Pearl Trilogy Imaging System. (c). Bar graph demonstrating fluorescence of flank tumors as compared to other organs. Bars indicate means with standard error bars.

To evaluate the biodistribution of VGT-309, we administered female athymic nude mice bearing flank OE19 xenografts measuring 250 ± 50 mm^3^ intravenous VGT-309 at a dosage of 2.0 mg/kg (*n* = 3 mice). After tail vein injection, the fluorescence of tumor and background tissue (contralateral flank) was recorded using the Vision Sense Imaging System (Figure 4(a)). We performed ex vivo organ biodistribution analysis at 24 hours using the Pearl Imaging System and showed that tumors displayed strong fluorescent signal (mean MFI: 172.4 A.U.) with fluorescent signal in the kidneys, liver, and spleen consistent with known cathepsin expression in these tissues and renal clearance of the free probe (Figures 4(b) and 4(c)). Of note, there was no fluorescent signal in the esophagus and stomach, consistent with known limited cathepsin expression in these tissues at baseline [20].

To determine if VGT-309 could label esophageal cancer xenografts, female BALB/c mice bearing AKR (murine ESCC) and HNM007 (murine ESCC) flank allografts and female athymic nude mice bearing OE19 (human EAC) flank xenografts (*n* = 3 per group) were administered 2 mg/kg VGT-309 via tail vein injection. Mice bearing KB (human cervical carcinoma) xenografts were used as a positive control, and those bearing KB xenografts pretreated with 50 mg/kg JPM-OEt via intraperitoneal injection daily for five days prior to VGT-309 administration were used as a negative control. Twenty-four hours after VGT-309 injection, mice were sacrificed and imaged with the Iridium NIR imaging system. The 24-hour time between infusion and imaging was chosen given prior murine studies evaluating VGT-309 imaging at this time point [21].

**Figure 5:**
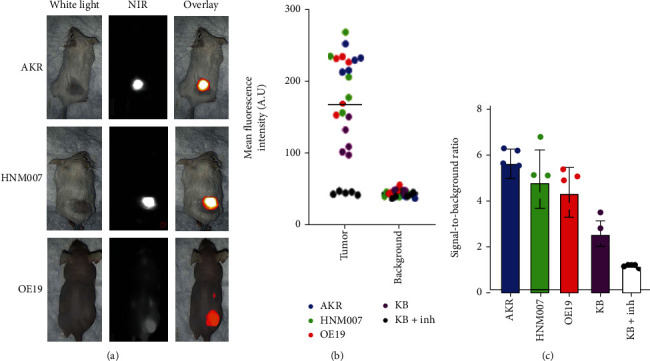
VGT-309 labels esophageal cancer allografts and xenografts. (a). Representative bright field, near-infrared (NIR) and overlay images of mice bearing various esophageal cancer flank tumors (*n* = 3/cell line) 24 hours after injection of 2 mg/kg VGT-309. Mice bearing KB flank xenografts (*n* = 3) pretreated with 50 mg/kg JPM-OEt via intraperitoneal injection for 5 days before VGT-309 administration served as a negative control and those pretreated with delivery vehicle (*n* = 3) served as a positive control. (b). Mean fluorescent intensity (MFI) of tumor and background tissue with symbol color indicating cell line. (c). Signal-to-background ratios (SBRs) from the flank tumors. Each point represents a single tumor with means and error bars displayed for each group.

Tumors derived from all esophageal cancer cell lines displayed strong macroscopic fluorescent signal (Figure 5(a)). Pretreatment of xenograft-bearing mice with a pan-cathepsin inhibitor nearly eliminated fluorescent signal from flank tumors, indicating cathepsin activity dependence of the fluorescent signal. On post hoc analysis of fluorescent images, the region of interest corresponding to flank tumors had significantly higher MFI than those corresponding to background tissue on the contralateral flank of the same mice (Figure 5(b), *p* < 0.0001 in all cases). There were no significant differences between MFI for tumor and background tissue in the mice pretreated with pan-cathepsin inhibitor. Mean SBR was greater than 2.0 for all esophageal cancer flank tumors, indicating excellent tumor-specific labeling (Figure 5(c)).

**Figure 6:**
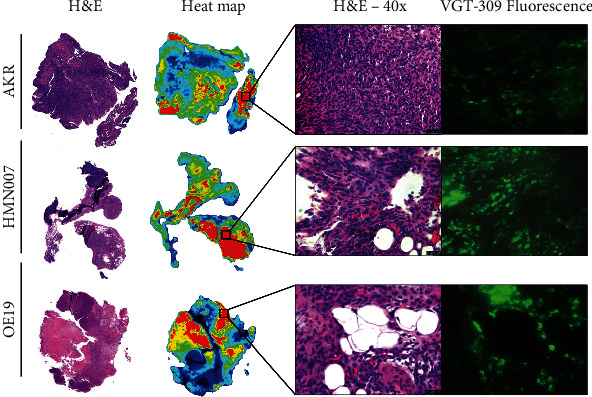
VGT-309 labels esophageal cancer flank tumors during resection. (a) Representative tissue sections from all three cell lines stained with hematoxylin and eosin and imaged with the Odyssey NIR imaging system. (b) High magnification microscopy images showing H&E staining and fluorescence microscopy from the same area of tumors.

Analysis of excised tumor sections using the Odyssey NIR imaging system showed that VGT-309 signal was distributed throughout the tumors derived from the three esophageal cancer cell lines (Figure 6). Microscopic analysis of tumors by hematoxylin and eosin staining as well as fluorescence microscopy confirmed VGT-309 signal on a cellular level.

## 4. Discussion

In this study, we evaluated VGT-309, a cathepsin-targeted, quenched activity-based NIR probe, for identifying esophageal cancer during resection. Using multiple preclinical models, we demonstrated that VGT-309 reliably labels both esophageal squamous cell carcinoma and esophageal adenocarcinoma during resection. Importantly, we found that normal esophageal and gastric tissue is not labeled by VGT-309, indicating that this tracer has significant potential for clinical translation in differentiating cancer from normal tissue during surgery. To our knowledge, this is the first study to test a covalent activity-based NIR probe during resection of esophageal cancer.

This work builds upon a robust body of preclinical literature evaluating the utility of cathepsin-targeted probes for imaging cancer. This has included many studies in cellular systems and animal models of cancer [21, 26–28]. Cathepsin substrate probes have been shown to label esophageal cancer in two preclinical evaluations in murine models of fluorescence endoscopy [20, 29]. More recently, a cathepsin substrate probe containing a far-red fluorophore, LUM015, was used to image human breast and sarcoma resection specimens [30]. Smith and colleagues evaluated the same probe in ex vivo breast cancer resection specimens and have also demonstrated its ability to detect of residual cancer when resection cavities were imaged [31, 32]. Because LUM015 requires a tracer-specific imaging device to detect signal in the far-red spectrum, its depth of penetration is limited to surface level tumors and its clinical adoption may be hindered by the fact that the imaging device for its detection is not widely available.

To improve depth of tumor detection and allow compatibility with existing FDA-approved clinical imaging devices, recent work has focused on designing cathepsin-targeted probes in the NIR spectrum [26, 33–35]. VGT-309 is based upon the parent probe BMV109, which was developed using a Cy5 fluorophore for use in cellular and animal models [27]. The probes contain the same phenoxymethyl ketone electrophile that covalently and irreversibly binds active cysteine cathepsins, but in VGT-309, the far-red Cy5 fluorophore is replaced with the near-infrared ICG [21]. The use of this near-infrared (NIR) fluorophore confers two major advantages: (1) significant depth of signal penetration to identify tumors below the organ surface and (2) the ability to be detected by FDA-approved, widely available surgical imaging devices.

Within the field of thoracic surgery, lung cancer has been the predominant focus of studies of intraoperative molecular imaging (IMI). Our group and others have demonstrated the ability of targeted and nontargeted tracers to localize nonpalpable tumors, quantify resection margin distances, identify synchronous metastases, and evaluate sentinel lymph nodes, among other indications [8–10, 36–39]. Significantly less attention has been given to the potential applications of IMI in surgery for esophageal cancer. Intraoperative imaging using ICG has primarily been used to assess perfusion of the gastric conduit and aid in sentinel lymph node identification [40]. Therefore, this study represents a major advance in the field of fluorescence-guided surgery as the first to demonstrate successful use of a qABP for guiding resection of esophageal cancer in real time.

Further studies are in progress to validate the utility of this technology in other preclinical models, and the ultimate test will be a human trial to fully characterize the benefits and limitations of VGT-309. Nonetheless, there is clear clinical promise of VGT-309 to localize esophageal cancer during surgery or during endoscopic surveillance in high-risk patients. Ultimately, this technology may provide benefit to patients in reducing the need for re-resection, lowering the rates of local recurrence, and personalizing adjuvant therapy for esophageal cancer.

## Data Availability

All data supporting the results of this study are available upon request from the corresponding author.
